# The *In* *Vitro* and *In* *Vivo* Fungal Volatile Organic Compounds Associated with Rapid ʻŌhiʻa Death and the Response of Xyleborine Ambrosia Beetles to those Compounds

**DOI:** 10.1007/s10886-025-01606-1

**Published:** 2025-05-27

**Authors:** Kylle Roy, Eva Brill, Dan Mikros, Kelsey Tobin, Jennifer Juzwik, Brandon Mcnellis, Douglass Jacobs, Lisa Keith, Dong H. Cha, Matthew Ginzel

**Affiliations:** 1 U.S.D.A. Forest Service, Forest Health Protection, Pacific Southwest Region, Hilo, HI USA; 2 Purdue University, Department of Forestry and Natural Resources, West Lafayette, IN USA; 3 U.S.D.A. Agricultural Research Service, Daniel K. Inouye U.S. Pacific Basin Agricultural Research Center, Hilo, HI USA; 4 University of Hawaiʻi at Hilo, Hawaiʻi Cooperative Studies Unit, Hilo, HI USA; 5 U.S.D.A. Forest Service, Northern Research Station, St. Paul, MN USA; 6 U.S.D.A. Agricultural Resource Services, Jornada Experimental Range, Las Cruces, NM USA; 7 Purdue University, Department of Entomology, West Lafayette, IN USA

**Keywords:** Fungal Volatiles, Chemical Signaling, *Ceratocystis*, Mutalistic Fungi, Behavior

## Abstract

Rapid ‘ōhiʻa death (ROD), caused by fungal pathogens *Ceratocystis lukuohia* and *Ceratocystis huliohia*, is devastating ʻōhiʻa (*Metrosideros polymorpha*), the keystone forest tree species of Hawaiʻi. Ceratocystidaceae fungi produce fruity volatile organic compounds (VOCs) and sticky sexual spores, suggesting they are adapted for insect dispersal. In this study, we explored chemical signaling of the causal agents of the ROD disease complex and how those VOCs mediate ambrosia beetle (Coleoptera: Curculiondiae: Scolytidae)-plant interactions. Four species of ambrosia beetles (*Xyleborinus saxesenii, Xyleborus affinis, Xyleborus ferrugineus*, and *Xyleborus perforans*) are direct vectors of the ROD pathogens in addition to spreading the fungi indirectly through contaminated frass. To further understand the extent to which semiochemicals mediate relationships between agents of the ROD pathosystem, we identified fungal VOCs both *in vitro* and *in vivo*. We then tested behavioral responses of the ROD-associated ambrosia beetles to the odor of the fungi using still-air olfactometer assays. Similar fruity and banana-like fusel alcohols and acetates were produced by *C. lukuohia* and *C. huliohia* in culture and when colonizing ‘ōhiʻa seedlings, which could be detected one-day post-inoculation, nine days before symptom onset. *X. affinis* was attracted to both *C. lukuohia* and *C. huliohia*, while *X. ferrugineus* positively responded to *C. lukuohia* and *X. perforans* to *C. huliohia*. These findings suggest a semiochemical association and potential support for the hypothesis of coevolutionary history between *Ceratocystis* fungi and insects.

**Supplementary Information:**

The online version contains supplementary material available at 10.1007/s10886-025-01606-1.

## Introduction

The most common ambrosia beetles cultivate and consume mutualistic fungi belonging to the *Ambrosiella* (Microascales: Ceratocystidaceae), *Fusarium* (Hypocreales: Nectriaceae), and *Raffaelea* (Ophiostomatales: Ophiostomataceae) genera (Kostovcik et al. 2015; Mayers et al. 2015; Wingfield et al. 2017; Ranger et al. 2021). These saprotrophic fungi are carried by beetles in specialized pocket-like structures called mycetangia (Hulcr et al. 2015; Vega and Biedermann 2020; Mayers et al. 2022) and are perhaps derived from ancestors that were adapted for cuticular dispersal on insects (Kostovcik et al. 2015; Mayers et al. 2015, 2022; Wingfield et al. 2017). There is a growing understanding that ambrosia beetles are attracted to volatiles produced by their mutualistic fungi and use olfactory cues to distinguish nutritional mutualists from other fungi (Hulcr et al. 2011; Martini et al. 2017; Ranger et al. 2021; Gugliuzzo et al. 2023).

The Ceratocystidaceae family contains many important tree pathogens and insect symbionts (de Beer et al. 2014). These fungi are adapted for insect dispersal based on their sexual structures and volatile organic compounds (VOCs) (Kile 1993; Wingfield et al. 2017). Their sexual structures or ascomata, known as perithecia, have extended sheaths that exude ascospores in a sticky matrix that facilitates acquisition by insects (Kile 1993; Wingfield et al. 2017). Dispersing insects may be attracted to volatiles of metabolic products of Ceratocystidaceae fungi, including fruity, banana, and floral odors composed of fusel alcohols and acetates (Hanssen 1993; Mailula et al. 2025).

Some of the most well-studied Ceratocystidaceae-insect dispersal systems include oak wilt, canker stain of plane, and rapid ʻōhiʻa death (ROD). Oak wilt kills *Quercus* spp., most prominently red oaks, in eastern North America and is caused by *Bretziella fagacearum*, primarily vectored by sap beetles (Coleoptera: Nitidulidae) in the Upper Midwest and Texas (Lin and Phelan 1992; Juzwik et al. 2004, 2011). Canker stain of plane, caused by *Ceratocystis platani* and vectored by the ambrosia beetle *Platypus cylindrus* (Curculionidae), is killing plane (*Platanus* spp.) trees in Europe (Tsopelas et al. 2017; Soulioti et al. 2015; Brilli et al. 2020). *Ceratocystis lukuohia* and *Ceratocystis huliohia* are the causal agents of rapid ʻōhiʻa death (ROD). These pathogens are vectored by ambrosia beetles and the disease complex is killing ʻōhiʻa lehua (‘ōhiʻa; *Metrosideros polymorpha*) trees in Hawaiʻi (Keith et al. 2015; Roy et al. 2023). However, the relationships among the insect vectors, fruiting bodies, and VOCs of Ceratocystidaceae fungi in these systems are not well understood, and lacking laboratory behavioral evidence.

Understanding the plant, beetle, and pathogen relationship of ROD is especially challenging due to the complexity of the two different *Ceratocystis* pathogens. *C. lukuohia* is more virulent than *C. huliohia*, where *C. lukuohia* manifests as a vascular wilt and *C. huliohia* as a canker disease, although outward symptoms may be similar (Hughes et al. 2020; Juzwik et al. 2024). *C. lukuohia* is in the Latin-American geographical clade (LAC) of *Ceratocystis*, which contains many aggressive tree pathogens, while *C. huliohia* is of the Asian-Australian clade (AAC) (Barnes et al. 2018). Population genetics suggests *C. lukuohia* may be a more recent introduction than *C. huliohia* (Barnes et al. 2018), and evidence supports horizontal gene transfer between the two species (Mayers et al. 2021). In addition, there are emerging patterns of *C. huliohia*-dominated forests being converted to *C. lukuohia* in Hawai’i (Brian Tucker, Pacific Cooperative Studies Unit, written communication, 2022), although the cause of this shift is unclear.

‘Ōhiʻa is the most bioculturally significant tree species in the Hawaiian archipelago (Abbott 1992; Mueller-Dombois et al. 2013), comprising 150,000 hectares of biomass (Gon et al. 2006). The ambrosia beetle vectors of *C. lukuohia* and *C. huliohia* are members of the Xyleborini (Coleoptera: Curculionidae: Scolytinae) tribe including *Xyleborinus* (*Xi.*) *saxesenii* (Ratzburg), *Xyleborus* (*X.*) *affinis* Eichhoff*, Xyleborus* (*X.*) *ferrugineus* (Fabricius)*,* and *Xyleborus* (*X.*) *perforans* (Wollaston). These beetles reproduce in ʻōhiʻa trees that are infested with ROD-*Ceratocystis* and carry these fungi on their exoskeletons (Roy et al. 2023); they may exacerbate co-infections and intensify spread through their frass which may contain viable, long-lived chlamydospores (Hughes et al. 2022; Roy et al. 2023).

Although *C. lukuohia* and *C. huliohia* are not likely the primary ambrosia fungi of the ROD-associated beetles, *X. affinis* and *X. ferrugineus* are also vectors of closely related *Ceratocystis* species (Saunders and Knoke 1967; Herrera and Grillo 1989; Goitía and Rosales 2001; Souza et al. 2013; Galdino et al. 2016; Romero et al. 2022), and congeneric fungi have been isolated from the mycetangia of a sister species (Kostovicik et al. 2015). We hypothesize that invasive ROD-associated beetles are attracted to ROD-*Ceratocystis* fungi, as these fungi may produce chemical signals that indicate habitat suitability and/or influence feeding behavior of the beetles (Davis et al. 2013).

The objectives of our study were to identify fungal VOCs associated with the ROD pathosystem and characterize the behavioral response these VOCs elicit in the ROD-associated beetles. We hypothesize that both ROD-*Ceratocystis* fungal pathogens in culture and ʻōhiʻa trees would produce short-chain VOCs that attract ROD vectors. To identify the VOCs of ROD, we initially isolated the headspace volatiles of *C. lukuohia* and *C. huliohia* in culture (*in vitro*). Subsequently, we inoculated ʻōhiʻa seedlings with both ROD pathogens to determine the change in VOCs emitted from the inoculation point over time, *in vivo*. Finally, we conducted still-air olfactometer assays with all four ROD-associated ambrosia beetles to determine their short-range behavioral responses to the ROD-*Ceratocystis* VOCs.

## Methods and Materials

### Fungal Culture Preparation for Volatile Analysis

We collected VOCs produced by four *C. lukuohia* isolates, four *C. huliohia* isolates, one *Ceratocystis* sp. isolate from *Syngonium*, and one *Ceratocystis uchidae* isolate from taro using solid-phase microextraction (SPME). *Ceratocystis* sp. of *Syngonium* previously reported in the Caribbean is the closest related species of *C. lukuohia* in the LAC and *C. uchidae* from Hawaiʻi and Fiji is closest to *C. huliohia* in the AAC (Barnes et al. 2018). Two of the *C. lukuohia* isolates were originally obtained from Hawaiʻi Island (P14-1–1 and C21-18) and two were from Kauaʻi (C21-16 and C20-13), Hawai’i, USA. Similarly, two of the *C. huliohia* isolates were originally collected from Hawaiʻi Island (P15-59 and P18-91) and two (C21-14 and P18-91) from Kauaʻi. The *Ceratocystis* isolate from *Syngonium* was originally obtained from Hawaiʻi Island and *C. uchidae* from Bun-long taro on Oʻahu, Hawaiʻi. All isolates, except *C. uchidae*, are actively maintained in the U.S. Department of Agriculture-Agricultural Research Service-Daniel K. Inouye-U.S. Pacific Basin Agricultural Research Center (USDA-ARS-DKI-PBARC) culture collection in Hilo, Hawaiʻi. We obtained the *C. uchidae* isolate from Dr. Thomas Harrington at Iowa State University. We grew cultures on 2% Potato Dextrose Agar (PDA) [19.5 g BD Difco™ Dehydrated Potato Dextrose Agar (Becton, Dickinson and Company, Franklin Lakes, New Jersey, USA) per 500 ml distilled water] in 60 × 15 mm plastic Petri dishes (Fisherbrand™, Fisher Scientific, Hampton, New Hampshire, USA) for 14 days at room temperature to ensure perithecial lawns (fungal growth throughout the culture plate) before inoculation on fungal slants. We selected PDA agar because it can support fungal growth to maturity (i.e., perithecia production; Mailula et al. 2025).

We prepared fungal slants based on Ranger et al. (2021), using 20 × 150 mm glass test tubes with threaded black phenolic screw caps (Thermo Fisher Scientific, Waltham, Massachusetts, USA). First, we removed the supplied plastic septa from screw caps and used a 5-mm drill bit to create an opening through which the SPME fiber could be inserted. Then we covered the opening with a 17-mm Advanced Green 3 septum (Chromatography Research Supplies, Louisville, Kentucky, USA), with the pre-bored hole facing out. We cleaned the vials, caps, and septa with Alconox® powdered precision cleaner (Alconox, White Plains, New York, USA), 70% ethanol, and 100% acetone and then baked them in a drying oven at 100 °C for 12 h prior to media preparation.

We prepared two-percent PDA media and pipetted 20 ml aliquots into glass test tubes. We then capped the tubes and placed them at a 20° angle and the media was allowed to solidify for at least one hr. Using a sterile loop, we inoculated the PDA in individual glass tubes with ascospores of the fungal isolates. Prior to chemical analysis, we incubated three replicates of each fungal isolate and three PDA controls at room temperature in the dark for 14 days, simulating natural growth within the xylem.

### Volatile Analysis of *Ceratocystis* in Culture

**Fig. 1:**
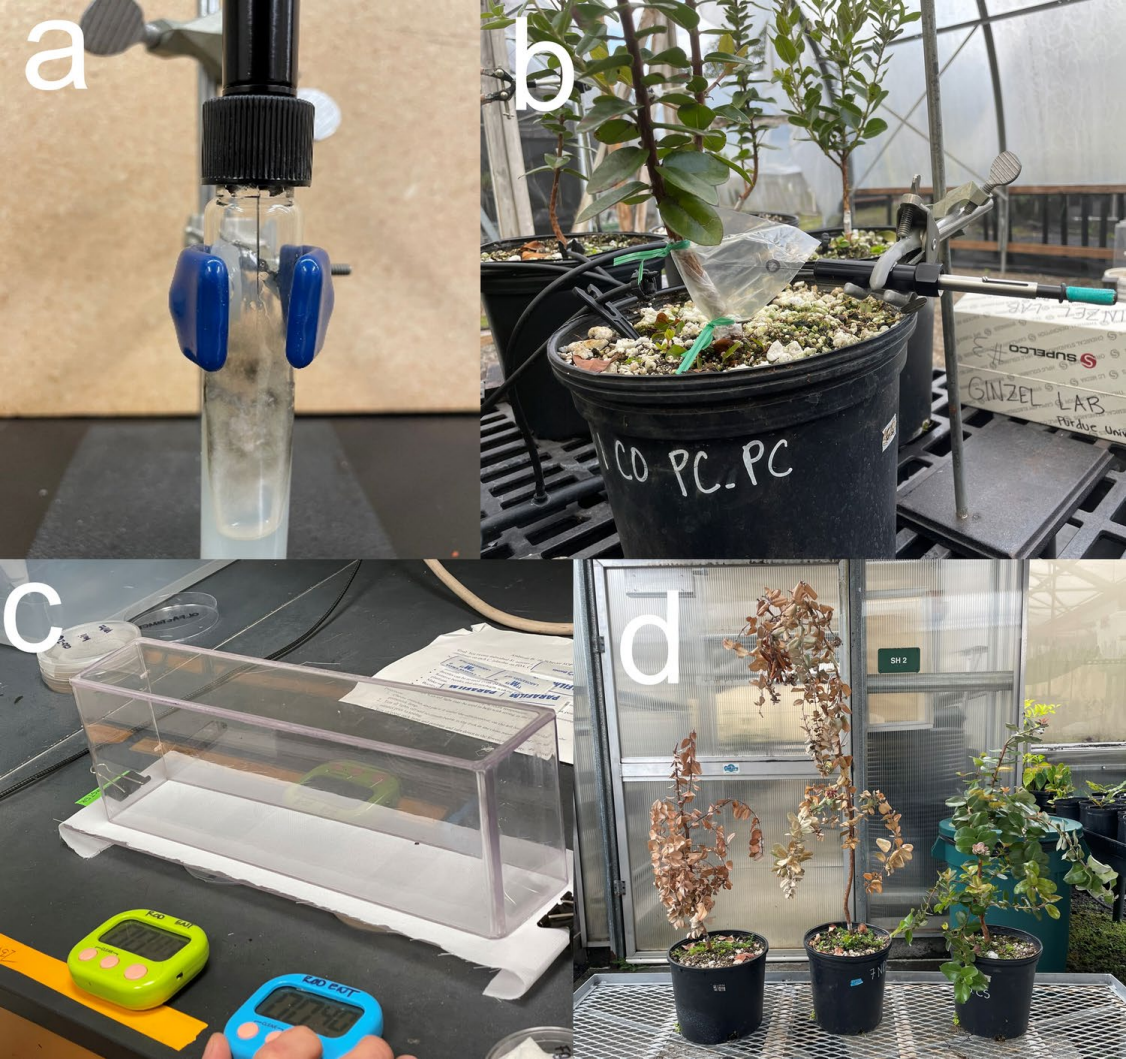
Experiments to determine the volatile organic compounds produced by rapid ʻōhiʻa death-*Ceratocystis* and the behavioral response of ambrosia beetle vectors to the fungi. **a)** Solid phase microextraction (SPME) technology was used to collect volatiles from the headspace of fungal slants. **b)** SPME was used to collect volatiles from plastic bags sealed around the inoculation point of ʻōhiʻa stems. **c)** A still-air olfactometer was used for bioassays to time ambrosia beetle response to fungal cultures grown in Petri dishes placed below the olfactometer. **d)** ʻŌhiʻa seedlings 45 days after inoculation, from left to right: inoculated with *C. lukuohia*, *C. huliohia*, and water as control. All photos by Kylle Roy

To characterize the volatiles produced by *C. lukuohia* and *C. huliohia* in a controlled environment, we used solid-phase microextraction gas chromatography-mass spectrometry (SPME GC–MS) to collect and identify volatile emissions from all *Ceratocystis* isolates grown on PDA media and PDA alone (control). Prior to volatile collections, we conditioned the SPME fiber (DVB/carboxen/PDMS, Sigma-Aldrich, St. Louis, Missouri, USA) for 10 min at 225 °C. A syringe containing a retracted SPME fiber was inserted through the septum of the glass tube slant and secured to a ring stand to position the fiber approximately 1 cm above the fungal culture (Fig. 1a). We exposed the SPME fiber to the headspace for 30 min and then immediately analyzed the sample by GC–MS.

We thermally desorbed the SPME fiber in an Agilent 6890 N GC (Agilent Technologies, Santa Clara, California, USA) gas chromatograph interfaced with an Agilent 5975B mass spectrometer with electron impact ionization (70 eV). The GC–MS was equipped with a SPME liner (0.75 mm ID, Supelco, Bellefonte, Pennsylvania, USA), a non-polar DB-5 ms capillary column (30 m × 0.25 mm × 0.25 µm film thickness; J&W Scientific, Folsom, California, USA) and helium carrier gas. We ran the samples under splitless mode (1 min sampling time) with an initial 40 °C hold for 1 min, followed by 1.5 °C/min ramp to 90 °C, 5 °C/min ramp to 150 °C, and 10 °C/min ramp to 250 °C.

We identified compounds unique to each fungal isolate and absent from the PDA control by comparing their mass spectra with those in the National Institute of Standards and Technology (NIST) mass spectral library (ca. 120,000 spectra; ChemStation Version D.05.01; Hewlett Packard Corp., Palo Alto, California, USA). We confirmed our identifications by matching spectra and retention times to those of pure standards (Sigma-Aldrich, St. Louis, Missouri, USA). When authentic standards were unavailable, we calculated Kováts retention indices (for propyl acetate and ethyl propionate) to confirm the identity of individual compounds. The relative abundance of each compound in extracts was calculated as a percentage of the total corrected peak area of all consistently identified compounds present in the total ion chromatograms. For each isolate, we calculated the mean percent abundance and standard error of the contribution of each compound to the total across all replicates.

### Seedling Inoculum Preparation and Procedure

To determine the influence of inoculation on VOC profiles over time, we used fifteen, two-year-old ʻōhiʻa seedlings (mean height 64.53 ± 3.86 cm; mean diameter at soil 10.73 ± 0.59 mm) grown in 7.57 L [2 gal] containers that were kept in a greenhouse with a drip irrigation system. We assigned five seedlings to each of three treatment groups: 1) inoculated with *C. lukuohia* isolate C21-16, 2) inoculated with *C. huliohia* isolate C20-2, and 3) water inoculated controls. We obtained both isolates from USDA-ARS-DKI-PBARC, with selection based on maintenance of morphological characteristics and recency since re-isolation from diseased ʻōhiʻa. Due to the time needed for SPME sampling and analysis, we inoculated no more than two replicates of each treatment at the same time. We conducted the experiment in four rounds. In the first three rounds, we inoculated one replicate of each treatment, and in the fourth round, we inoculated two replicates of each treatment, resulting in a total of five replicates per treatment.

We used the paper disc inoculation technique as described in Keith et al. (2015) with minor modifications. Briefly, we added 2 ml of sterile water to 7–10-day-old cultures grown on v8 media [50 ml V8® vegetable juice, 0.10 g calcium carbonate, 10 g agar, per 500 ml distilled water] and scraped the surface with a rubber policeman. We pipetted 10 µl of the liquid into a hemocytometer and calculated the number of spores twice before diluting to a 10^6^ spore/ml suspension. We soaked sterile paper discs (10 mm diameter) in the diluted spore suspension and then inoculated them onto V8 media plates, incubating at 25 ºC for 4–5 days.

Following preparation, we inoculated stems of ʻōhiʻa seedlings by using a sterile scalpel to make two alternating 15–20 mm incisions at the potline (4.23 ± 0.20 cm above the soil line, 10.40 ± 0.43 mm diameter at inoculation point) of each seedling, exposing the sapwood. We then placed a paper disk in the incision and added an additional 20 µl of liquid inoculum to the incision. Finally, we then carefully repositioned the outer bark over each wound and secured it with all-purpose laboratory film (Parafilm®, Amcor, Yakima, Washington, USA).

### Inoculated Seedling Volatile Collection and Analysis

We collected fungal volatiles emitted at inoculation sites on the ʻōhiʻa seedlings at 0, 1, 3, 10, 21, 30, and 45 days after inoculation (DAI) using SPME followed by GC–MS analysis. We wrapped the inoculation site of each seedling air-tight with a modified, 7.6 × 7.6 cm, chemically inert plastic bag (see Stewart-Jones & Poppy 2006; Reynolds Consumer Products LLC, Lake Forest, Illinois, USA) and a twist-tie. Prior to volatile collections, we conditioned the SPME fiber (DVB/carboxen/PDMS, Sigma-Aldrich, St. Louis, Missouri, USA) for 10 min at 250 °C. We positioned the SPME holder using a clamp attached to a ring stand and pierced the SPME needle through the plastic bag, exposing the SPME fiber for one hr to capture volatiles in the headspace (Fig. 1b). Immediately following the collection period, we thermally desorbed the SPME fiber at 250 ºC in the injection port of an Agilent 7890B GC (Agilent Technologies, Santa Clara, California, USA) gas chromatograph interfaced with an Agilent 5977 A mass spectrometer. The GC–MS inlet was equipped with a SPME liner (0.75 mm ID, Supelco, Bellefonte, Pennsylvania, USA), a polar DB-WAXETR capillary column (30 m × 0.25 mm × 0.25 µm film thickness; Agilent Technologies, Santa Clara, California, USA), and helium carrier gas. We ran samples under splitless mode with an initial hold for 5 min at 40 °C, followed by a 15 °C/min ramp to 250 °C and 5 min hold at 250 °C. We identified compounds unique to each fungal isolate and absent from the PDA control using NIST library searches. We confirmed our identifications by matching their mass spectra and retention times with those of pure standards from Sigma-Aldrich. When authentic standards were unavailable, we confirmed the identity of individual compounds by calculating Kováts retention indices, particularly for propyl acetate. We calculated and reported the relative abundance of these compounds as described above.

### Inoculated Seedling Disease Severity Assessment

On each day of volatile collection, we rated the severity of disease symptoms on a 0 to 5 scale, where 0 = no symptoms, 1 = 1–25% of leaves wilted, 2 = 26–50% wilt, 3 = 51–75% wilt, 4 = 76–99% wilt, 5 = 100% (Fig. 1d). After the final SPME collection, we transported the seedlings to the lab and harvested them by first removing the branches with a sterile clipper, leaving the main stem. We debarked the main stem with the sterile blade of a grafting knife and assessed for vascular discoloration above and below the inoculation point. We report the average percent vascular discoloration, above and below the inoculation point, relative to the average height of the seedlings. To confirm the presence and identity of the pathogen, we removed thin wood sections of the main stem at 3 cm and 10 cm above the inoculation point and performed DNA and viability analysis as described in Roy et al. (2023).

### Ambrosia Beetle Behavioral Bioassay

We used a still-air walking olfactometer to assess the behavioral arrestment responses (as a proxy of attraction) of all four invasive ROD-associated ambrosia beetles to cultures of *C. lukuohia* and *C. huliohia* grown on 2% PDA, and negative controls containing only 2% PDA. For comparison, we also assessed the response of beetles to another fungus, the biological control agent, *Beauvaria bassiana* (provided by USDA-ARS-DKI-PBARC). We used the same fungal isolates as those used for seedling inoculations (i.e., *C. lukuohia* isolate C21-16 and *C. huliohia* isolate C20-2) in behavioral bioassays. We grew all cultures on 100 × 15 mm sterile polystyrene Petri dish (Fisherbrand™, Fisher Scientific, Waltham, Massachusetts, USA) 2% PDA plates for 14 days under ambient tempterature prior to use. All cultures used in bioassays were between 14–20 days old to ensure consistent volatile profiles and individual cultures were used for a single day of assays.

We modified the methods described by Ranger et al. (2021) for conducting the behavioral assays using a still-air olfactometer. In brief, we placed a plexiglass acrylic chamber (30 × 6 × 10.5 cm; L x W x H) with the open side down over an acrylic platform with two openings (5 × 2.5 cm) covered with fine mesh polyester fabric (35 × 35 squares/cm^2^) (Fig. 1c). Individual beetles were contained in the chamber and the mesh-covered openings were placed directly over an empty Petri dish blank control or the tested stimulus (fungal culture or PDA negative control), allowing the beetle to choose between odor sources. We conducted a minimum of 60 trials per beetle species for each stimulus treatment.

Female beetles were either reared from ROD-*Ceratocystis* infested bolts or trapped using cross vane panel traps (CVPT; Forestry Distributing, Boulder, Colorado, USA) as in Roy et al. (2023) at the Komohana Research and Extension Center, Hilo, HI, an area with an active ROD-*Ceratocystis* outbreak. Once collected, we immediately identified beetles to species under a dissection microscope and then placed the beetles in a Petri dish lined with a damp paper towel at room temperature near a natural light source (to avoid disrupting their circadian rhythm). Individual beetles were utilized for assays once per 24 h and no more than five days post-collection. We only used beetles that were apparently healthy and active in bioassays.

All female beetles used in bioassays were brought into the testing room and allowed to acclimate to the environment for at least 30 min prior to testing. We placed a single beetle into the center of the olfactometer and subjected her to a 5-min assay. The total time a beetle spent over the mesh-covered rectangular opening above either stimulus or control was recorded. The time spent over stimuli is henceforth referred to as arrestment response. We conducted bioassays from 13:00 to 18:00 HST, the approximate peak active period of other Xyleborine species (Ranger et al. 2021). Beetles that did not begin walking or showing signs of activity within 30 s of being transferred to the platform were removed and the trial discarded. We applied the same protocol to beetles that primarily spent their time flying with minimal time on the platform. We alternated the placement of the control blank and stimulus every four trials, such that each stimulus would be tested evenly on both sides of the olfactometer. After every four trials, or between stimuli or beetle species, the olfactometer was thoroughly cleaned with warm soapy water, rinsed, and dried with paper towels.

### Statistical Analyses

For the inoculated seedlings, we examined the influence of DAI and inoculation type (*C. lukuohia* or *C. huliohia)* on wilt symptoms by conducting generalized linear mixed models (GLMM) with DAI and treatment as fixed effects and a random effect of replicate. We focused VOC analyses on acetates found in both *C. lukuohia* and *C. huliohia*-inoculated seedlings including ethyl acetate, isobutyl acetate, and isoamyl acetate, as these compounds produce fruit-like odors hypothesized to support co-evolution. To assess the relationship of acetate abundance emitted from seedlings with inoculation type and DAI, we used similar GLMMs, with DAI and treatment as fixed effects, and a random effect of replicate. We logit transformed all acetate abundance data (Warton and Hui 2011). Preliminary analysis indicated a strong quadratic polynomial response in VOC abundance over time in some sample groups. To account for this, we added an interaction term that grouped each DAI into either an “early infection” or “late infection” category and allowed the relationship between VOC abundance and DAI to change in sign after 10 DAI. Models were fit using the Gaussian response family and identity link. We assessed overall model fit based on residual diagnostic plots and performance metrics of simulated residuals (Zuur et al. 2009). We further fit models with inflection points at 3 DAI and compared them to our 10 DAI model using Akaike Information Criterion (AIC) scores.

For the olfactometry bioassays, beetle arrestment responses (i.e. attraction) to PDA, *C. lukuohia*, *C. huliohia*, or *B. bassiana* were compared to those elicited by the blank Petri dish controls for each beetle species using Mann–Whitney U tests for non-parametric comparisons. To compare the arrestment response times of beetle species to the four different stimuli, we used Kruskal–Wallis rank sum tests, with significant differences followed by pairwise Wilcox tests for multiple pairwise comparisons.

We used R version 4.5.0 (R Core Team 2022) using the R packages ‘tidyverse’ (Wickham et al. 2019), ‘cowplot’ (Wilke 2020), ‘emmeans’ (Length 2021), ‘glmmTMB (Brooks et al. 2017), and ‘DHARMa’ (Hartig 2022) to analyze all data, defining statistical significance at *α* = 0.05.

## Results

### Fungal Volatiles from Cultures

We detected nine VOCs from *C. lukuohia* and ten from *C. huliohia* in culture*,* all of which were ester alcohols or acetates. Both species produced (in at least one isolate) the following compounds: ethyl acetate, 2-methyl-1-propanol (isobutanol), ethyl propionate, propyl acetate, 3-methyl-1-butanol (isoamyl alcohol), 2-methly-1-butanol, isobutyl acetate, 3-methylbutyl acetate (isoamyl acetate), 2-methylbutyl acetate, and 2-phenylethanol (Table 1). Of note, ethyl acetate and isobutyl acetates produce general fruity odors and isoamyl acetate produces a banana aroma. We detected only trace levels of phenyl ethyl alcohol, in one *C. lukuohia* isolate and two *C. huliohia* isolates. Interestingly, *C. huliohia* consistently emitted ethyl propionate, a compound with a pineapple-like odor, which was absent in volatile collections from *C. lukuohia*. VOC abundance varied by isolate, in cultures of both *C. lukuohia* and *C. huliohia*. The volatile profiles of both conspecific fungi tested, *Ceratocystis* sp. of *Syngonium* and *C. uchidae*, resembled those of *C. huliohia*, though they lacked isobutanol and 2-phenylethanol (Table 1). When comparing *C. lukuohia* to *Ceratocystis* sp. of *Syngonium* and *C. uchidae*, the conspecific fungi lacked both isobutanol and 2-phenylethanol in addition to ethyl propionate.

**Table 1:** Mean percent abundance (± SE) of volatile organic compounds (VOCs) determined by solid phase microextraction (SPME) from four *C. lukuohia* isolates, four *C. huliohia* isolates, and one isolate of the most closely related species to each rapid ‘ōhiʻa death causal agent. VOCs were collected from each isolate in replicates of three, 14-day-old cultures grown on 2% PDA media. The percent of the total compounds in each replicate was averaged across isolates (nd = not detected; values in parentheses indicate retention time)

Fungal species	Isolate	Collection Location	ethyl acetate (1.77)	isobutanol (1.80)	ethyl propionate (2.64)	propyl acetate (2.68)	isoamyl alcohol (3.00)	2-methyl-1-butanol (3.04)	isobutyl acetate (3.83)	isoamyl acetate (7.06)	2-methyl-butyl acetate (7.16)	2-phenylethanol (22.65)
*Ceratocystis lukuohia*	P14-1–1	Leilani, Hawaiʻi	4.24 ± NA	nd	nd	nd	nd	31.25 ± 8.44	32.41 ± 29.1	7.89 ± 1.19	5.42 ± 0.34	0.48 ± NA
	C21-18	Hawaiʻi National Park, Hawaiʻi	35.10 ± 32.81	11.32 ± 0.79	nd	nd	nd	80.00 ± 2.02	5.37 ± 1.06	0.66 ± 0.09	2.26 ± NA	nd
	C20-13	Hanalei, Kauaʻi	4.38 ± 0.83	8.03 ± 1.60	nd	0.79 ± NA	nd	39.74 ± 15.63	35.64 ± 12.97	6.61 ± 2.43	7.11 ± 2.55	nd
	C21-16	Kōkeʻe, Kauaʻi	3.68 ± 1.20	7.91 ± 1.38	nd	nd	53.93 ± NA	41.18 ± 1.16	22.47 ± 10.68	4.51 ± 1.63	2.27 ± 1.01	nd
*Ceratocystis huliohia*	P15-59	Hōlualoa, Hawaiʻi	6.31 ± 1.22	7.17 ± 1.34	0.53 ± 0.06	1.07 ± NA	40.24 ± NA	28.25 ± 5.77	32.42 ± 3.90	6.09 ± 0.52	4.55 ± 0.51	0.35 ± 0.11
	P18-91	Kailua, Hawaiʻi	13.90 ± 1.89	nd	0.22 ± 0.03	0.98 ± 0.09	nd	11.90 ± 4.93	59.96 ± 5.50	6.83 ± 1.40	6.19 ± 1.08	nd
	C21-14	Moloaʻa, Kauaʻi	4.80 ± 1.25	5.48 ± 1.59	0.64 ± 0.24	0.734 ± NA	46.76 ± 4.10	25.02 ± 6.77	24.95 ± 5.14	3.85 ± 0.92	3.177 ± 0.63	0.88 ± 0.48
	C20-2	Lihue, Kauaʻi	5.33 ± 1.12	6.12 ± 1.54	0.51 ± 0.17	0.87 ± NA	44.19 ± 6.83	24.79 ± 3.95	25.64 ± 6.87	5.54 ± 1.59	4.35 ± 1.49	nd
Syngonium *Ceratocystis* sp.	P16-1	Panaʻewa, Hawaiʻi	16.62 ± 2.40	nd	0.16 ± 0.07	1.37 ± 0.10	11.01 ± 4.23	14.87 ± 5.52	34.38 ± 1.54	15.00 ± 1.21	10.31 ± 0.66	nd
*Ceratocystis uchidae*	C1714	Honolulu, Oʻahu	8.09 ± 0.24	nd	0.66 ± 0.26	4.64 ± 0.60	23.47 ± NA	23.56 ± 7.68	46.35 ± 0.77	5.54 ± 0.38	3.34 ± 0.07	nd

### Fungal Volatiles from Inoculated Seedlings

When we inoculated ‘ōhiʻa seedlings with either *C. lukuohia* or *C. huliohia*, we detected the same major compounds produced by corresponding fungal cultures, except for ethyl propionate and propyl acetate, which were present only in *C. huliohia* cultures (Tables 2, 3). The only VOC we detected from water-inoculated control seedlings was ethanol which is a known tree stress compound. Specifically, we detected ethanol from three of the control seedlings 3 DAI, then in single replicates for the remainder of the experiment. We also detected ethanol in the *C. lukuohia* and *C. huliohia* inoculated seedlings, also beginning at 3 DAI and persisting throughout the experiment (Tables 2, 3).

**Table 2:** Mean percent abundance (± SE) of fungal volatile organic compounds determined by solid phase microextraction (SPME) from ‘ōhiʻa seedlings (*N* = 5) inoculated with *C. lukuohia* isolate C21-16 over time. The percent of the total compounds in each seedling was averaged across treatment (DAI = Days after inoculation; nd = not detected)

Compound	Retention Time	1 DAI	3 DAI	10 DAI	21 DAI	30 DAI	45 DAI
ethyl acetate	2.86	nd	0.31 ± 0.14	1.56 ± 1.33	3.14 ± 1.12	nd	nd
ethanol	3.51	nd	0.21 ± NA	1.44 ± 1.26	7.64 ± 0.16	9.37 ± NA	100 ± 0
propyl acetate	4.32	nd	0.33 ± NA	0.03 ± NA	nd	nd	nd
isobutyl acetate	5.30	80.19 ± 19.81	97.82 ± 0.92	93.56 ± 2.94	76.36 ± 3.74	57.32 ± NA	nd
isobutanol	7.12	nd	2.26 ± 0.87	4.47 ± 1.14	14.71 ± 3.83	59.55 ± 18.18	100 ± NA
isoamyl acetate and 2-methlybutyl acetate	7.59	nd	1.63 ± 0.78	1.71 ± 0.69	6.66 ± 0.94	11.03 ± NA	nd
Isoamyl alcohol and 2-methyl-1-butanol	8.92	nd	0.03 ± NA	0.04 ± NA	nd	8.02 ± 7.23	nd
2-phenylethanol	15.53	39.61 ± NA	nd	nd	nd	nd	nd

The retention times for isoamyl acetate and 2-methylbutyl acetate as well as isoamyl alcohol and 2-methyl-1-butanol on the polar column were similar, producing overlapping peaks, therefore the same abundance is reported for both

**Table 3:** Average percent abundance (± SE) of fungal volatile organic compounds determined by solid phase microextraction (SPME) from ‘ōhiʻa seedlings (*N* = 5) inoculated with *C. huliohia* isolate C20-2 over time. The percent of the total compounds in each seedling was averaged across treatment (DAI = Days after inoculation; nd = not detected)

Compound	Retention Time	1 DAI	3 DAI	10 DAI	21 DAI	30 DAI	45 DAI
ethyl acetate	2.86	100	nd	2.83 ± 2.62	4.97 ± 00	nd	nd
ethanol	3.52	nd	1.83 ± NA	0.24 ± 0.03	0.81 ± 00	100	100
isobutyl acetate	5.30	nd	72.43 ± 5.29	90.45 ± 2.29	78.39 ± 9.03	nd	nd
isobutanol	7.13	nd	4.46 ± 7.12	6.79 ± 1.81	25.30 ± 10.01	100	100
Isoamyl acetate and 2-methylbutyl acetate	7.57	nd	nd	0.22 ± 0.09	1.95 ± 00	nd	nd
Isoamyl alcohol and 2-methyl-1-butanol	8.93	nd	nd	0.44 ± 0.42	nd	nd	nd
2-phenylethanol	15.53	nd	9.14 ± 15.53	0.20	nd	nd	nd

The retention times for isoamyl acetate and 2-methylbutyl acetate as well as isoamyl alcohol and 2-methyl-1-butanol on the polar column were similar, producing overlapping peaks, therefore the same abundance is reported for both

**Fig. 2:**
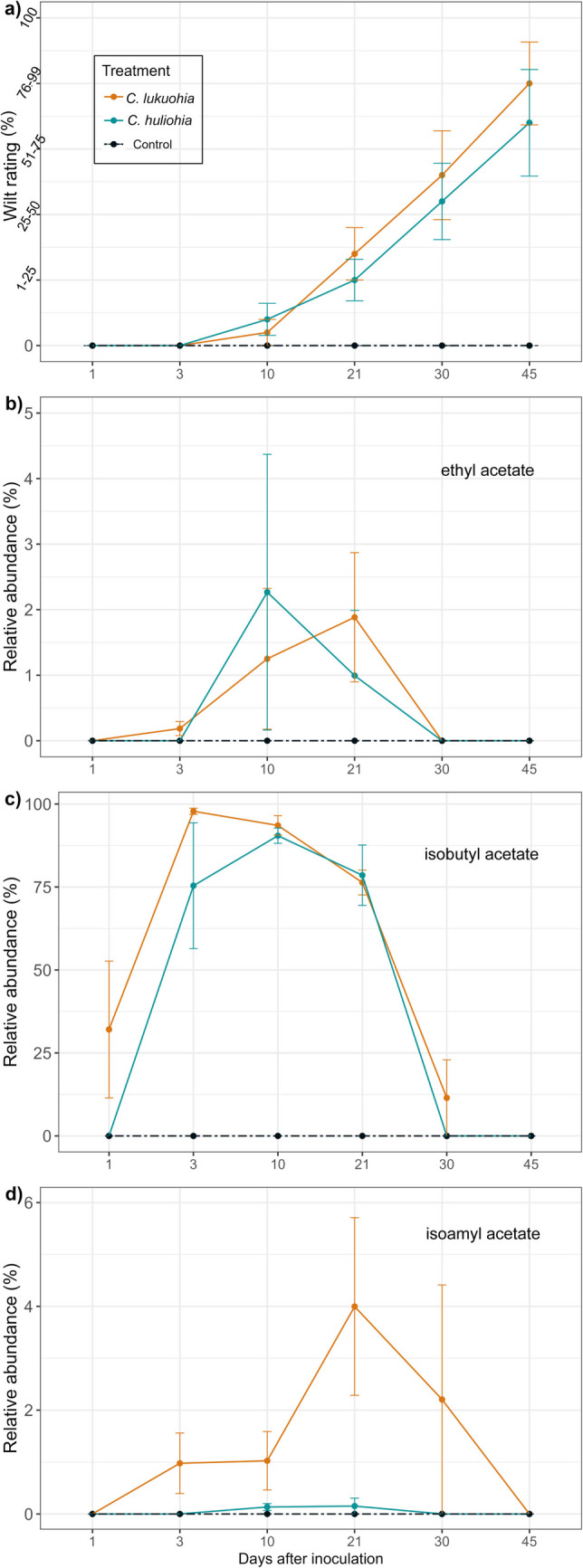
Mean (± SE) wilt rating **a**) and **b-d**) relative abundance of fungal volatile organic compounds detected at the inoculation point of ‘ōhiʻa seedlings infected with *C. lukuohia*, *C. huliohia*, or water paper disc controls, over time

The relative abundance of ethyl acetate did not differ between ‘ōhiʻa seedlings inoculated with *C. lukuohia* or *C. huliohia* (Table S1; Fig. 2b). However, isobutyl acetate significantly increased until 10 DAI, then began to decrease in both *C. lukuohia and C. huliohia*-inoculated seedlings (Table S2;Fig. 2c). *C. lukuohia*-inoculated seedlings emitted higher levels of isoamyl acetate compared to negligible levels for those inoculated with *C. huliohia* (Table S3; Fig. 2d).

### Disease Assessment Over Time

We first observed wilting symptoms at 10 DAI in both *C. lukuohia* and *C. huliohia*-inoculated seedlings, but fungal VOCs, including ethyl acetate, isobutyl acetate, and phenylethyl alcohol, were detected as early as 1 DAI (Tables 2, 3,Fig. 1d; 2a, b, d). We found no differences in the rates of wilt symptoms between *C. lukuohia* and *C. huliohia,* and wilt symptoms increased greatly after 10 DAI onward (Fig. 2a;Table S4).

Both *C. lukuohia* and *C. huliohia* DNA and viable *Ceratocystis* propagules were consistently present in samples from 1 cm above the inoculation point of all *Ceratocystis*-inoculated seedlings. However, at 10 cm above the inoculation point, 80% of the seedlings inoculated with *C. lukuohia* contained fungal DNA and viable propagules, while *C. huliohia* was not detected at 10 cm (Table 4). No ROD-*Ceratocystis* DNA or viable propagules were detected in the water-inoculated control seedlings. Vascular discoloration corresponded to the presence of DNA and viability. In seedlings inoculated with *C. lukuohia*, 26% of their length above the inoculation point showed staining, compared to only 11% of those inoculated with *C. huliohia*. We observed minimal staining, less than 1% of the total stem, in water-inoculated controls.

**Table 4:** Frequency of ROD-*Ceratocystis* detection and relative extent of vascular discoloration observed in ʻōhiʻa seedlings 45 days after inoculation with *Ceratocystis lukuohia*, *Ceratocystis huliohia*, or water controls. Isolates were confirmed genetically by qPCR and morphologically on carrots from samples taken 1 cm above the inoculation point (IP) and 10 cm above the IP

Inoculum	Pathogen presence 1 cm above IP^1^		Pathogen presence 10 cm above IP^1^		Percent total stem height with vascular discoloration^2^	
	DNA	Culture	DNA	Culture	Below IP	Above IP
*C. lukuohia*	5	5	4	4	4	26
*C. huliohia*	5	5	0	0	3	11
Water control	0	0	0	0	< 1	< 1

^1^Five seedlings were inoculated with each type of inoculum

^2^Stem heights were on average, 64.53 ± 3.86 cm for the inoculated seedlings. The percent vascular discoloration is based on the length of discoloration in relation to the IP divided by total stem height multiplied by 100

#### *Beetle Response to Fungal Volatiles Xi. saxesenii*

**Fig. 3:**
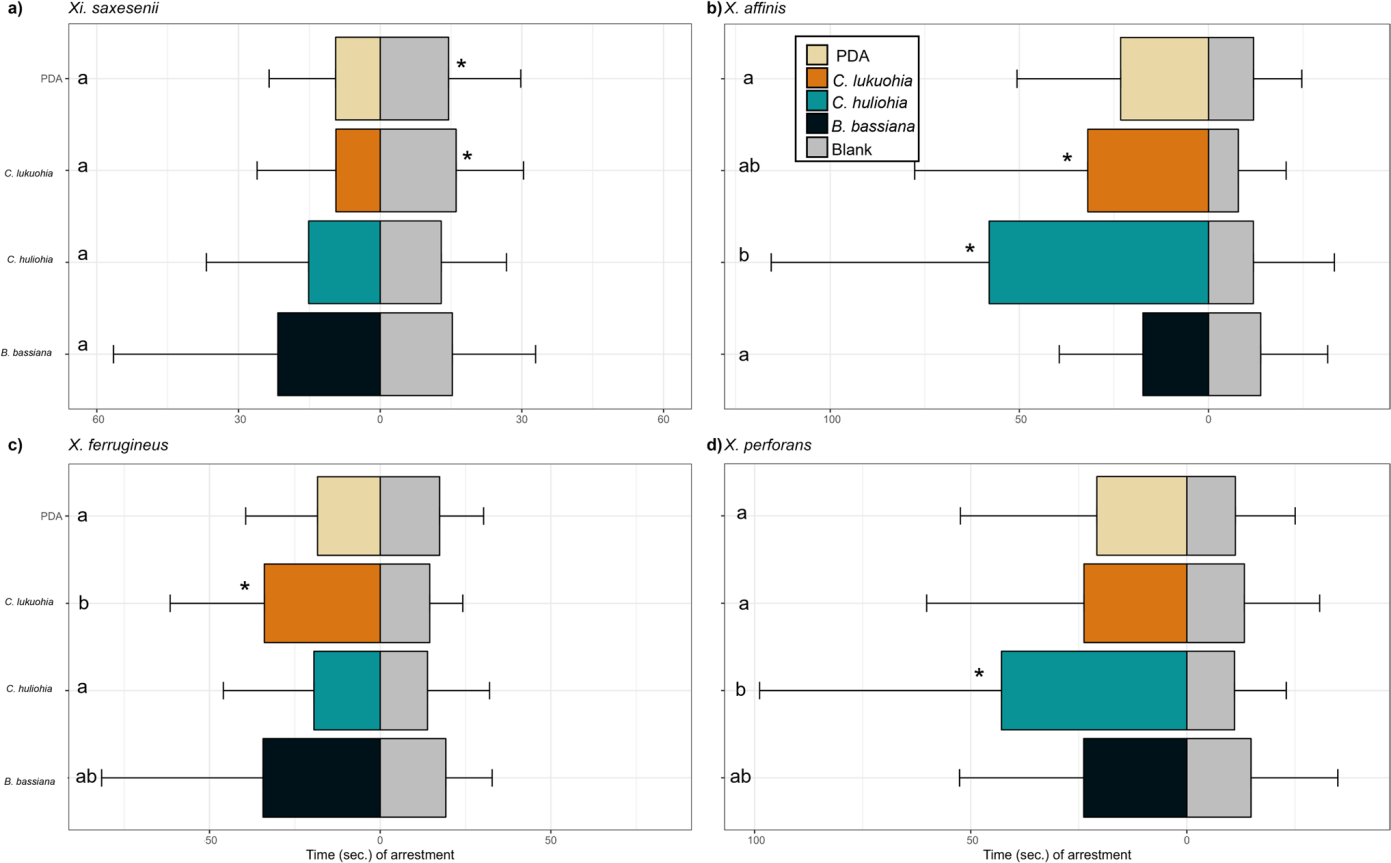
The mean arrestment response (± SD) of **a)** *Xi. saxesenii*, **b)** *X. affinis*, **c)** *X. ferrugineus*, and **d)** *X. perforans* ambrosia beetles to volatiles emitted by PDA media, *C. lukuohia*, *C. huliohia*, *B. bassiana*, and blank Petri dish controls. Stimuli are colored bars on the left and blank controls are grey bars on the right. A total of 60 female beetles were tested for each combination of volatile stimulus source (*N* = 60). Significant differences are denoted by * for Mann–Whitney U tests between volatile stimuli and blank control arrestment responses and different letters indicate significant differences among the four stimuli arrestment responses according to Kruskal–Wallis rank sum tests

displayed a short-range arrestment response to the blank controls compared to *C. lukuohia* and PDA (*W* = 1178, *P* < 0.001; *W* = 1394, *P* = 0.03, respectively). However, there was no difference in *Xi. saxesenii* response to neither *C. huliohia* nor *B. bassiana* compared to the blank control (*W* = 1761, *P* = 0.83; *W* = 1781, *P* = 0.92, respectively; Fig. 3a). Additionally, the response of *Xi. saxesenii* did not differ among stimuli (*H*(2) 4.75, *DF* = 3, *P* = 0.19).

*X. affinis* exhibited a greater arrestment response to *C. lukuohia* and *C. huliohia* when compared to the blank control (*W* = 2839, *P* < 0.001; *W* = 3094, *P* < 0.001, respectively), and there was no difference in the response to *B. bassiana* and PDA media when compared to the blank control (*W* = 1864.5, *P* = 0.99; *W* = 2169.5, *P* = 0.05, respectively; Fig. 3b). The arrestment response of *X. affinis* varied among stimuli, with the longest response observed for *C. huliohia*, which differed from that to PDA and *B. bassiana*, (*H*(2) 23.71, *DF* = 3, *P* < 0.01). In contrast, the response to *C. lukuohia* was statistically similar to all other stimuli.

Volatiles of *C. lukuohia* elicited arrestment in *X. ferrugineus* compared to the blank control (*W* = 2525.5, *P* < 0.001). However, compared to the blank controls, there were no differences in the responses to PDA, *C. huliohia*, and *B. bassiana* (*W* = 1633, *P* = 0.38; *W* = 1796, *P* = 0.76; *W* = 1863.5, *P* = 0.99, respectively; Fig. 3c). Among stimuli, *C. lukuohia* volatiles elicited a longer arrestment from *X. ferrugineus* than did PDA or *C. huliohia* (*H*(2)13.86, *DF* = 3, *P* < 0.01), while the response to *B. bassiana* was statistically similar to the other stimuli.

*Xyleborus perforans* preferred *C. huliohia* over the blank control (*W* = 2839, *P* < 0.001), and there were no differences in arrestment times between PDA, *C. lukuohia*, or *B. bassiana* compared to the blank control (*W* = 2055.5, *P* = 0.16; *W* = 1798.5, *P* = 0.31; *W* = 2042.5, *P* = 0.20; respectively; Fig. 3d). Among stimuli, *X. perforans* spent more time over *C. huliohia* than *C. lukuohia* and PDA (*H*(2) 8.50, *DF* = 3, *P* = 0.04), while the response to *B. bassiana* did not differ from the other two.

## Discussion

We report here, for the first time, specific VOCs associated with ROD pathogens, both *in vitro* and *in vivo*, and the behavioral responses they elicted in the four invasive ROD-associated ambrosia beetles*.* Although VOC profiles of these two fungi showed minimal variation, three ROD-associated ambrosia beetles displayed attraction towards one or both of the fungi; this response suggests a ROD-*Ceratocystis*-insect association. In particular, *X. ferrugienus* responded strongest toward *C. lukuohia* while *X. affinis* and *X. perforans* exhibited the strongest attraction for *C. huliohia*.

We detected fusel alcohols and acetates that produce fruity and banana-like odors in *C. lukuohia* and *C. huliohia* in both laboratory cultures and in ROD-*Ceratocystis* infected live plants. These compounds are also common to other Ceratocystidaceae, such as the causal agents of oak wilt and canker stain of plane (Lin and Phelan 1992; Soulioti et al. 2015). Characterization of these VOCs could aid in the development of new tools (such as electronic noses and trained detector dogs) for early detection of ROD, a critical management need, particularly for *C. lukuohia*, as symptoms often only appear after a tree has advanced crown symptoms (Hughes et al. 2020). Detection of VOCs in seedlings as early as one day post-inoculation, nine days before any apparent symptoms, underscores their potential application for early, pre-visual detection of the disease. However, this rapid shift in VOC profiles following inoculation needs to be verified in older, field-grown trees.

Outward wilt symptoms manifested similarly in both *C. lukuohia* and *C. huliohia* seedlings (Fig. 2d). However, these symptoms may differ in fully mature forest trees, and were notably initially different in field-inoculated trees in Hughes et al. (2020) and Juzwik et al. (2024). Interestingly, the DNA and viability seedling results reflect the contrasting colonization patterns of *C. lukuohia* and *C. huliohia*. Specifically, *C. lukuohia* showed rapid systemic movement and was present along the length of the stem of nearly all samples. In contrast, *C. huliohia* exhibited more localized colonization and was not detected in samples taken 10 cm above the inoculation point (Table 4). Vascular discoloration caused by *C. huliohia* was on average, less than half the stem length compared to the greater extent of discoloration associated with *C. lukuohia* in inoculated seedlings.

Ethyl propionate, a pineapple-like scent, was the single compound unique to *C. huliohia* in culture, though it was not detected in inoculated seedlings. This discrepancy may be attributed to SPME fibers having varying affinities for different classes of compounds based on the coating material used or the compound may not have been captured effectively when other compounds were present. Interestingly, the most closely related *Ceratocystis* species to *C. lukuohia* (i.e., the one on *Syngonium*) and to *C. huliohia* (i.e., *C. uchidae*) also produced ethyl propionate (Table 1). Moreover, we observed higher abundance of isoamyl acetate in *C. lukuohia* than *C. huliohia*, (Fig. 2c, d), which could be evidence of a stronger banana-like odor in *C. lukuohia* than *C. huliohia*. The ethanol production from the control ʻōhiʻa seedlings was likely a stress signal, as ethanol is a product of anaerobic respiration (Ranger et al. 2018); these data support the hypothesis that ROD-associated ambrosia beetles are attracted to wounded and/or stressed ‘ōhiʻa (Roy et al. 2023). We expected *C. lukuohia* and *C. huliohia* to produce ethanol in culture, as Ranger et al. (2021) and Kuhns et al. (2014) found that related fungi produce ethanol. However, ethanol was not recovered from *C. lukuohia* and *C. huliohia* cultures (Table 1) but, when sampled with the same polar column used for the seedlings, ethanol was present (data not shown). Due to experimental differences, it is unclear whether the ethanol detected from the *C. lukuohia* and *C. huliohia*-inoculated seedings were produced from the seedlings or the fungi, or both.

Several ambrosia beetles are attracted to the scent of their mutualistic fungi (Hulcr et al. 2011; Ranger et al. 2021; Gugliuzzo et al. 2023) and ROD-associated beetles in the present study likely have mutualistic relationships with Ophiostomatalean *Raffaelea* fungi (Batra 1967; Harrington et al. 2010; Huclr et al. 2011). Related Ophiostomatalean fungi produce isobutyl acetate, isoamyl alcohol, isobutyl alcohol (Brilli et al. 2020), and isoamyl acetate (Kuhns et al. 2014), similar to compounds found in *C. lukuohia* and *C. huliohia*. In addition, *Ceratocystis* spp. have been sequenced from the mycetangia of *Xyleborini* beetles (Kostovicik et al. 2015), although the presence of ROD-*Ceratocystis* in mycetangia of these beetles has not been reported to date.

Our behavioral data suggest a positive relationship between *Ceratocystis* and insects, particularly with invasive ROD-associated beetles responding to volatiles emitted by these fungal pathogens (*X. affinis* and *X. ferrugineus* positively responded to *C. lukuohia* and *X. affinis* and *X. perforans* to *C. huliohia).* Both *X. affinis* and *X. ferrugineus* were attracted to *C. lukuohia* (Fig. 3b, c). These beetles are also implicated as vectors of *Ceratocystis fimbriata* (Souza et al. 2013; Galdino et al. 2016; Romero et al. 2022) and *Ceratocystis cacaofunesta* (Saunders et al. 1967; Goitía and Rosales 2001; Romero et al. 2022;), respectively, and both species are closely related to *C. lukuohia* (Barnes et al. 2018).

The neotropical origins of *X. affinis*, *X. ferrugineus,* and the *Ceratocystis* species in the LAC*,* may explain recurring insect-*Ceratocystis* associations, as both beetles displayed a preference for *C. lukuohia*. Of note, both *X. affinis* and *X. perforans* were attracted to the AAC species, *C. huliohia* (Figs. 3b, 2d). This is noteworthy for *X. perforans,* in particular, which is of tropical Asian origin (Roy et al. 2020). Despite the ability of *Xi. saxesenii* to inhabit and reproduce in *C. lukuohia*-infested ʻōhiʻa trees (Roy et al. 2020), our results suggest *Xi. saxesenii* may be repelled by *C. lukuohia* as well as PDA alone (Fig. 3a). This avoidance may be due to volatiles from the PDA medium itself, necessitating further research to resolve the relationship between *Xi. saxesenii* and ROD. In addition, the relatively prolonged and variable arrestment of *X. ferrugineus* and *X. perforans* over *B. bassiana* could be due to the presence of isoamyl alcohol and 2-methyl-1-butanol in the volatile profile of this entomopathogen (Ranger et al. 2021), compounds both abundant in those of *C. lukuohia* and *C. huliohia* (Fig. 3c;Tables 2–4). The nearly significant response of *X. affinis* to PDA could be addressed through assays testing its response to fungal stimuli grown on PDA versus PDA alone (instead of a blank Petri dish; Fig. 3d).

The identification of both *in vitro* and *in vivo* ROD fungal VOCs lays the groundwork for further testing the response of ambrosia beetles to these compounds, including evaluating the bioactivity of individual synthetic compounds and blends of ROD-*Ceratocystis* volatiles both in the lab and in the field. These compounds, along with host plant semiochemicals, are also likely key factors that mediate their colonization of ‘ōhiʻa trees and may be considered in further research. Of note, other beetle-vectored pathogens in the orders *Ophiostomatales* and *Hypocreales* alter the metabolic volatile profiles of host trees, directly influencing the behavior of their insect vectors (Nones et al. 2022; Tobin et al. 2025). This present study sheds light on the possible evolutionary dynamics of *Ceratocystidaceae* and their insect vectors, whereby fungal VOCs may serve as reliable infochemicals, indicating habitat suitability and influencing host selection behavior of the beetles (Davis et al. 2013).

## Supplementary Information

Below is the link to the electronic supplementary material.

Supplementary file1 (DOCX 16 KB)

## Data Availability

All relevant datasets are available upon request.
