# Supplementary material for: The In Vitro and In Vivo Fungal Volatile Organic Compounds Associated with Rapid ʻŌhiʻa Death and the Response of Xyleborine Ambrosia Beetles to those Compounds
Source: J Chem Ecol. 2025 May 27;51(3):59. doi: 10.1007/s10886-025-01606-1 (PMC12116855; doi:10.1007/s10886-025-01606-1)
Supplement: Supplementary file 1 — Supplementary file1 (DOCX 16 KB) [file 10886_2025_1606_MOESM1_ESM.docx]

**Supplementary Tables**

**Table S1.** Estimated coefficients from a generalized linear mixed model of inoculation type and days after inoculation (DAI) on ethyl acetate abundance.

| Effect | *β* ± SE | *z-*statistic | *P* |
| --- | --- | --- | --- |
| *C. huliohia* (Intercept) | 0.697 ± 0.421 | 1.658 | 0.097 |
| *C. lukuohia* | 0.009 ± 0.457 | 0.021 | 0.982 |
| DAI | -0.008 ± 0.015 | -0.572 | 0.567 |

**Table S2.** Estimated coefficients from a generalized linear mixed model of inoculation type and days after inoculation (DAI) on isobutyl acetate abundance, up to and after 10 days post-inoculation.

| Effect | *β* ± SE | *z-*statistic | *P* |
| --- | --- | --- | --- |
| *C. huliohia* (Intercept) | 0.413 ± 0.291 | 1.420 | 0.156 |
| *C. lukuohia* | 0.275 ± 0.259 | 1.063 | 0.288 |
| DAI: Infection Stage Up to 10 DAI | 0.240 ± 0.045 | 5.342 | <0.001* |
| DAI: Infection Stage After 10 DAI | -0.007 ± 0.009 | -0.795 | 0.426 |

**Table S3.** Estimated coefficients from a generalized linear mixed model of inoculation type and days after inoculation (DAI) on isoamyl acetate abundance.

| Effect | *β* ± SE | *z-*statistic | *P* |
| --- | --- | --- | --- |
| *C. huliohia* (Intercept) | -6.381 ± 0.272 | -23.419 | <0.001* |
| *C. lukuohia* | 0.894± 0.282 | 3.158 | <0.01* |
| DAI | -0.006 ± 0.009 | -0.612 | 0.541 |

**Table S4.** Estimated coefficients from a generalized mixed model of inoculation type and days after inoculation (DAI) on wilt rating, up to and after 10 days post-inoculation.

| Effect | *β* ± SE | *z-*statistic | *P* |
| --- | --- | --- | --- |
| *C. huliohia* (Intercept) | -0.305 ± 0.258 | -1.181 | 0.238 |
| *C. lukuohia* | 0.200 ± 0.229 | 0.872 | 0.383 |
| DAI: Infection Stage Up to 10 DAI | 0.053 ± 0.400 | 1.340 | 0.180 |
| DAI: Infection Stage After 10 DAI | 0.084 ± 0.008 | 10.295 | <0.001* |
